# A Case of Constipation During Cobenfy Therapy and a Constipation Prevention Protocol

**DOI:** 10.7759/cureus.105113

**Published:** 2026-03-12

**Authors:** Evaristus C Ezema, Amir Meftah, Bashir Aribisala, Satwant Singh, Tania Sultana, Jude Beauchamp, Uchenna Ezenagu, Thant Htet, Tolu Olupona

**Affiliations:** 1 Psychiatry, Interfaith Medical Center, Brooklyn, USA; 2 Psychiatry and Behavioral Sciences, Interfaith Medical Center, Brooklyn, USA

**Keywords:** cobenfy, constipation, constipation prevention protocol, prevention, protocol

## Abstract

Cobenfy is a new medication for the treatment of schizophrenia. It consists of a muscarinic agonist called xanomeline and a muscarinic antagonist called trospium. However, the associated gastrointestinal side effects, especially constipation, have a potential effect on compliance. Currently, there is no widely standardized constipation prevention protocol during Cobenfy therapy. We present a case of constipation during the treatment of schizoaffective disorder with Cobenfy in a middle-aged female. We conducted a literature review on Cobenfy therapeutic management strategies and provide constipation prevention strategies.

## Introduction

Cobenfy is a brand name for a new medication, pharmacologically called xanomeline-trospium [1]. In September 2024, the Food and Drug Administration (FDA) approved it for the treatment of schizophrenia in adult patients. This is a landmark advancement in the therapeutic management of schizophrenia as Cobenfy is not a traditional antipsychotic that usually works on dopamine receptors. The advancement of treatments for schizophrenia has been largely influenced by the necessity to create medications that exhibit more acceptable side-effect profiles. Schizophrenia is a chronic, severe brain disorder that causes individuals to interpret reality abnormally, often resulting in hallucinations, delusions, and disorganized thinking. In the 1950s, first-generation antipsychotics were introduced, which work primarily by acting as potent antagonists that block dopamine receptors in the brain [1]. The side effects range from extrapyramidal symptoms to metabolic syndrome [2]. The search for medications with fewer side effects resulted in the introduction of second-generation antipsychotics that work on serotonin and dopamine receptors [1]. Schizophrenia most often takes a chronic course, requiring long-term treatment with dopamine-blocking medications. The side effects of these medications continue to affect the patients’ compliance profile [2]. Even clozapine, which acts on serotonin, dopamine, and muscarinic receptors and is regarded as a gold standard for resistant schizophrenia treatment because of its efficacious effect on treatment-resistant schizophrenia, has its own significant side effects [3]. This concern led to continued interest in developing treatment options, leading to the development of Cobenfy.

Pharmacology

The mechanism of action of xanomeline is through binding to muscarinic receptors, i.e., M1 through M5 receptors [4]. However, the antipsychotic effect is through the agonistic action on M1 and M4 [3]. Xanomeline is reported to demonstrate a more agonistic affinity for M4 receptors than M1 [5]. Trospium is used together with xanomeline to mitigate the peripheral side effects of xanomeline at muscarinic receptors [6]. It has antagonistic properties at muscarinic receptors, potentially causing constipation and urinary retention [6]. Trospium is equipotent across muscarinic receptor subtypes [7]. As a polar quaternary amine, trospium cannot pass through the blood-brain barrier, thereby minimizing side effects on the central nervous system [7].

Xanomeline achieves maximum plasma concentration in 2.5 hours [8]. The bioavailability of xanomeline is poor (<1%) because of the extensive first-pass metabolism [8]. Hence, Cobenfy, an oral formulation, is taken twice daily, preferably on an empty stomach, one to two hours before eating or two hours after a meal to increase absorption and bioavailability [7]. Xanomeline has a wide tissue distribution, including the central nervous system [7]. Excretion of the drug is through the kidneys within 24 hours of administration [8]. Trospium chloride attains peak plasma concentrations in five to six hours following oral administration. This should occur an hour before meals or two hours after meals, as concurrent food ingestion significantly decreases trospium bioavailability. Negligible metabolism of trospium chloride occurs in the hepatic cytochrome P450 system, while excretion is mainly through the renal route [8]. Cobenfy is typically administered at a dose of 50 mg xanomeline/20 mg trospium, with a potential increase to 100 mg xanomeline/20 mg trospium [9]. Individuals with stomach outlet obstruction, liver diseases, urinary retention, hypersensitivity to xanomeline and/or trospium, and untreated narrow-angle glaucoma should avoid the use of Cobenfy [1]. Additionally, individuals with renal impairment (estimated glomerular filtration rate: <60 mL/minute) should not use Cobenfy [1].

Currently, there is no standardized constipation prevention protocol during Cobenfy therapy. Given this lack of a standardized constipation prevention protocol, we aim to present a case of constipation during Cobenfy therapy and propose a constipation prevention protocol. This study seeks to contribute to further understanding of Cobenfy therapy and provides clinical insights that may inform both management strategies and future research.

## Case presentation

We present the case of a 46-year-old African American female, single, with no children, a high school graduate, currently domiciled in supportive housing, currently unemployed, supported by government assistance with a long-standing history of schizoaffective disorder, depressive type, diagnosed 10 years ago, with more than four hospitalizations in the past, and a medical diagnosis of type 2 diabetes mellitus. The patient was admitted as a case of schizoaffective disorder, depressive type, on acute exacerbation. She was being managed in the psychiatric unit and had a daily bowel movement on monitor. Her physical examination during the intake and at the unit showed vital signs within normal limits, with stable blood pressure, heart rate ranging from 68 to 97 beats per minute, normal temperature, and normal respiratory rate. Oxygen saturation ranged between 95% and 98% on room air. Laboratory investigations revealed a complete blood count with differentials and a metabolic panel within reference ranges, as shown in Table 1 and Table 2, except for blood sugar. The fasting blood sugar levels were initially high, above 300 mg/dL (reference range: <100 mg/dL) during the first three days, but they were gradually controlled with a sliding scale of insulin regimen.

**Table 1:** Complete blood count.

Blood component	Patient’s value	Reference range
White blood cells	6,500/mm^3^	4,500–11,000/mm^3^
Red blood cells	4.1 million/mm^3^	4.3–5.9 million/mm^3^
Hemoglobin	13.6 g/dL	12.0–15.5 g/dL
Hematocrit	39%	36–44%
Mean corpuscular volume	86 fL	80–100 fL
Mean corpuscular hemoglobin	27 pg/cell	27–33 pg/cell
Mean corpuscular hemoglobin concentration	32%	32–36%
Platelets	250,000/mm^3^	150,000–450,000/mm^3^

**Table 2:** Complete metabolic panel. ALP = alkaline phosphatase; ALT = alanine aminotransferase; AST = aspartate aminotransferase; BUN = blood urea nitrogen

Component	Patient’s value	Reference range
Sodium	139 mEq/L	135–145 mEq/L
Potassium	4.1 mEq/L	3.5–5.2 mEq/L
Chloride	100 mEq/L	96–106 mEq/L
CO_2_ (bicarbonate)	25 mEq/L	23–29 mEq/L
BUN	15 mg/dL	6–20 mg/dL
Creatinine	0.9 mg/dL	0.6–1.3 mg/dL
Calcium	9.1 mg/dL	8.5–10.2 mg/dL
Glucose (fasting)	300 mg/dL	<100 mg/dL
Albumin	4.1 g/dL	3.4–5.4 g/dL
Total protein	6.4 g/dL	6.0–8.3 g/dL
ALP	46 U/L	20–130 U/L
ALT	15 U/L	4–36 U/L
AST	12 U/L	8–33 U/L
Bilirubin	1.0 mg/dL	0.1–1.2 mg/dL

**Figure 1:**
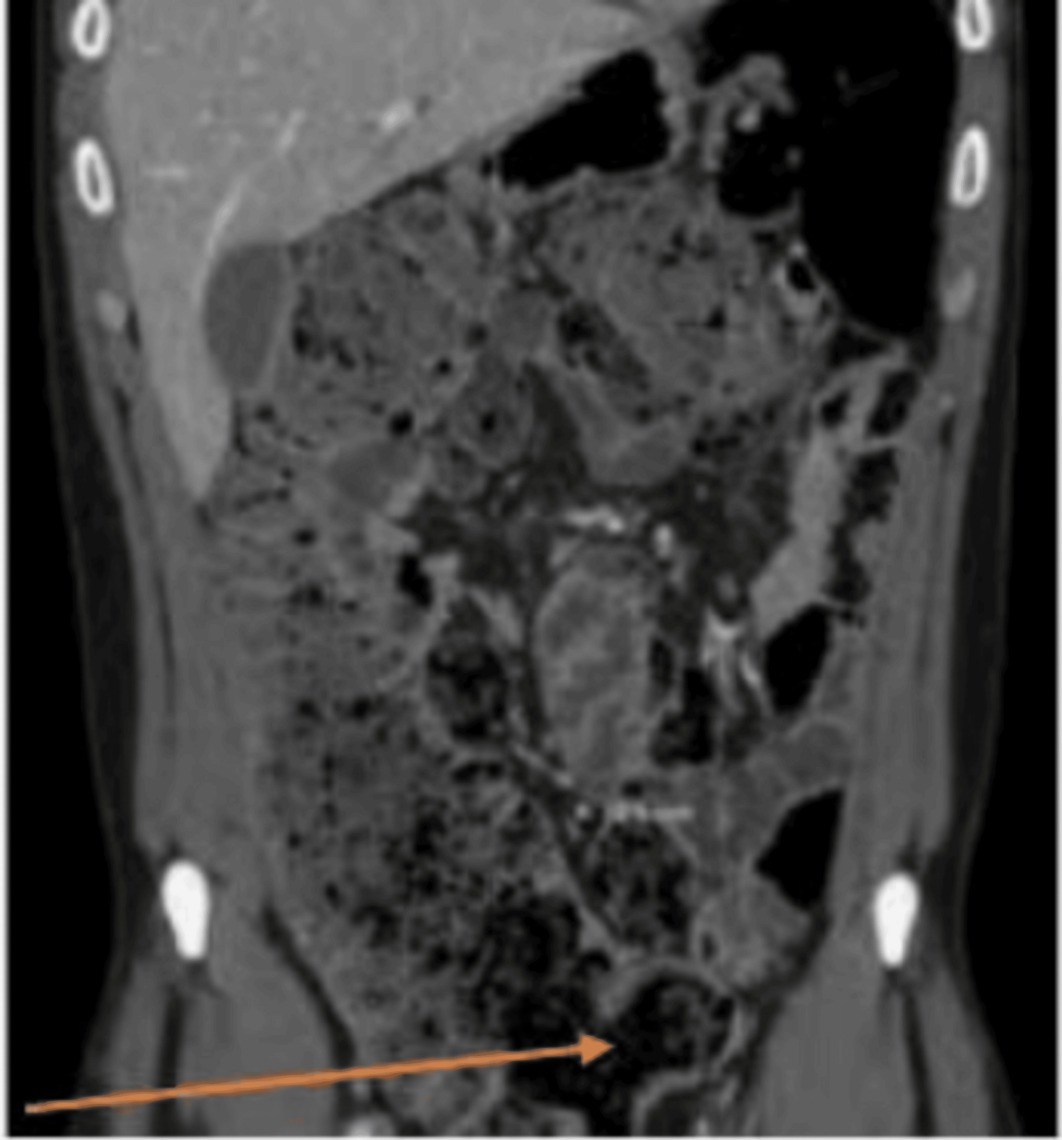
CT of the abdomen before treatment. The arrow shows the fecal retention.

**Figure 2:**
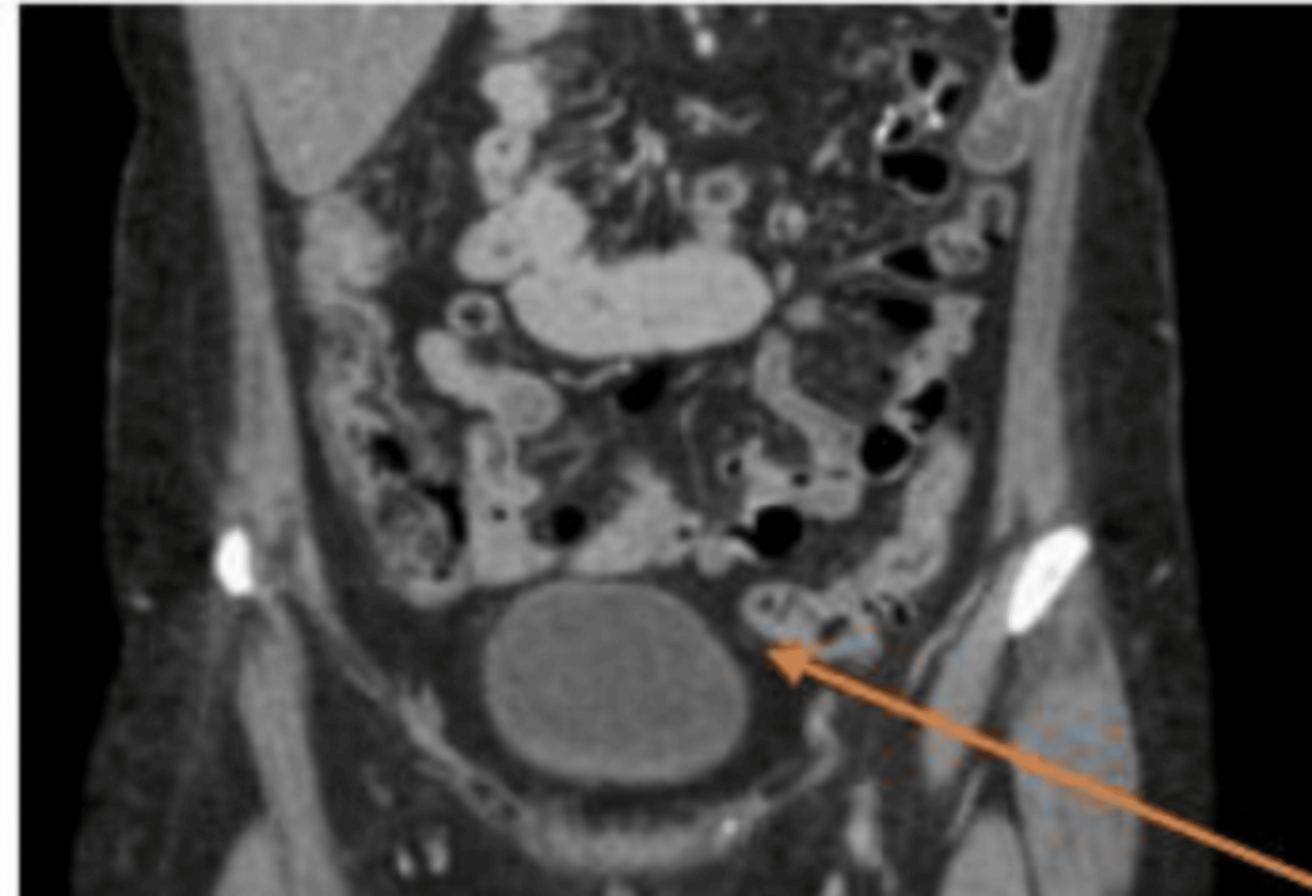
CT of the abdomen after treatment. The arrow shows the gut with no fecal retention.

A urine toxicology screen was negative for illicit substances, and the blood ethanol level was negative. The only initial radiological investigation was an unremarkable EKG. The clinical evaluation was remarkable for speech latency, disorganized thought process with looseness of association, and tangentiality. The patient was initiated on her usual home medications without dose adjustment, including fluphenazine 5 mg PO bid for psychosis, bupropion 300 mg PO daily for depression, and lamotrigine 100 mg PO bid for mood disorder. The patient was compliant with the medications, and a daily bowel movement was observed. After one week of treatment, the patient was not showing clinical improvement with persisting psychotic behaviors. The treatment team evaluated the patient and took a clinical decision to start her on Cobenfy. The patient was counseled on the benefits and risks along with the potential side effects of Cobenfy therapy, including constipation. The patient was started on Cobenfy 50/20 mg PO bid. She was also continued on home medications without dose adjustment, but on day four, no bowel movement was observed. There was no change in the patient’s usual diet. The next day, the patient was clinically evaluated by the medical team and started on polyethylene glycol 3350, 17 g daily until bowel movement. As the patient had no significant psychiatric improvement, on the third day of constipation, Cobenfy was discontinued, and the patient was transferred to the medical floor. On the medical floor, there was no change in the patient’s diet. A supine and upright view of the abdominal CT scan showed marked colonic fecal retention, as shown in Figure 1. Polyethylene glycol 3350, 17 g, was increased to bid, and lactulose 20 g daily was commenced. The patient eventually underwent a rectal enema, and a colonic pack of feces was removed. Constipation eventually resolved on day four, and the patient was transferred back to the psychiatric unit, where she continued on fluphenazine 5 mg PO bid for psychosis, bupropion 300 mg PO daily for depression, and lamotrigine 100 mg PO bid for mood disorder, her home medications. A repeat abdominal CT no longer showed fecal retention, as shown in Figure 2. Eventually, the patient showed clinical improvement with a logical and organized thought process. She was subsequently discharged after nine weeks of inpatient care. She was scheduled for a follow-up at the outpatient clinic for medication management, including initiation of long-acting injectables and psychotherapy in the following week.

## Discussion

In the central nervous system, cholinergic projections originate from two primary regions of the brain. The connection from the pedunculopontine-lateral dorsal tegmentum of the brain stem extends to the thalamus, midbrain, and brainstem, as well as the basal forebrain nuclei, which project to the limbic system and cerebral cortex [10]. The striatum and nucleus accumbens also contain cholinergic interneurons [10]. The metabotropic G-protein coupled receptors (GPCRs) are muscarinic, with a steady response on stimulation [11]. They are classified into two different functional receptor classes based on coupling to different GPCR subtypes, whereas nicotinic receptors are ligand-gated ion channels that respond quickly [11]. Different amounts of muscarinic receptors can be found throughout the body; the brain has a strong expression of M1, M4, and M5, while the periphery primarily contains M2 and M3 [12]. The frontal cortex, hippocampus, laterodorsal tegmental nucleus, and striatum are among the brain regions that have high concentrations of M1 and M4 receptors and are crucial for dopamine modulation [13]. A decrease in dopamine release in the ventral tegmental region may result from the activation of post-synaptic M1 receptors on GABA interneurons [13]. Pre-synaptic M4 autoreceptor stimulation reduces acetylcholine release, which, in turn, reduces dopamine release in the ventral striatum and nucleus accumbens [14].

These receptors, especially M1 and M4, have been exploited in novel drug invention, given their benefits in relieving psychosis and enhancing cognition [15]. These receptors in individuals with schizophrenia are the current targets for schizophrenia medications, including Cobenfy [15].

It is important to remember that individuals with schizophrenia, such as the index case, frequently have a disorganized thought process, negative symptoms, and reduced verbal expression. This could be linked to either a challenge or an incapacity to completely understand or express feelings or distress [16]. In this instance, the patient’s minimally conversant behavior limited the scope of the mental status examination. The treatment team monitored the patient’s bowel movement for concern about Cobenfy’s potential side effects and therefore kept a defecation log.

The index case illustrates a good case for the therapeutic application of Cobenfy. The patient had chronic schizoaffective disorder and had a long history of antipsychotic therapy that acts on dopamine receptors. It has been reported that long-term use of antipsychotic agents downregulates dopamine receptors [17]. The chronic use of antipsychotics acting on dopamine receptors might have accounted for frequent psychotic crises in the index patient. However, compliance outside the acute setting is difficult to ascertain. As early as the late 2000s, xanomeline single therapy showed efficacy in improving symptoms in schizophrenic patients and those with a diagnosis of schizoaffective disorder [18]. Cobenfy is described as a new frontier in schizophrenic management in adults and older adults [19]. Therefore, its application in the treatment of the index patient was highly advocated. However, there is no fully established protocol to prevent side effects arising from the use of Cobenfy. The side effects commonly observed include nausea, indigestion, constipation, vomiting, hypertension, and gastroesophageal reflux disease [9].

Untreated constipation arising from anticholinergic effects can progress to death, especially when complications of constipation, such as necrotizing colitis, intestinal ischemia, intestinal necrosis, and abdominal distention leading to volvulus, are not treated [20]. The FDA heightened an existing warning that constipation caused by the schizophrenia medication can, uncommonly, develop into significant bowel problems in a communications statement made in 2020 [20].

The FDA’s earlier concern led to constipation prevention strategies during clozapine therapy. The strategies include advocacy to drink considerable amounts of water and other liquids, get enough exercise, and eat more fruits, vegetables, and fiber-rich grains. Additionally, it is recommended that the patient be put on a laxative [20].

In view of the foregoing, we recommend that counseling for commencement of Cobenfy therapy should be accompanied by a constipation prevention protocol. Our recommended protocol should first start with medication education about the potential constipation associated with Cobenfy therapy. Second, the patient should be taught the early signs of constipation. Third, they should be educated on the need for a daily log of defecation. Fourth, lifestyle modifications such as increasing fiber in the diet and daily exercise (if possible) should be instituted. Furthermore, laxatives should be started once Cobenfy is commenced.

## Conclusions

Cobenfy is the currently approved medication for schizophrenia with no direct effects on the dopaminergic receptors, thereby minimizing the side effects arising from dopamine receptor activities. Cobenfy’s efficacy has been documented with recognizable side effects. Therefore, psychiatrists should promote regular monitoring for these side effects, especially constipation, in schizophrenic patients. It becomes much more necessary in cases with a disorganized thought process, as in the index case. This report seeks to contribute to strengthening further understanding of Cobenfy therapy, potential side effects, and provide a constipation prevention protocol for Cobenfy therapy that may inform optimum schizophrenia management strategies and future research.

## References

[1] Smith CM, Augustine MS, Dorrough J, Szabo ST, Shadaram S, Hoffman EO, Muzyk A (2025). Xanomeline-trospium (Cobenfy(TM)) for schizophrenia: a review of the literature. Clin Psychopharmacol Neurosci.

[2] Huhn M, Nikolakopoulou A, Schneider-Thoma J (2019). Comparative efficacy and tolerability of 32 oral antipsychotics for the acute treatment of adults with multi-episode schizophrenia: a systematic review and network meta-analysis. Lancet.

[3] Siskind D, Siskind V, Kisely S (2017). Clozapine response rates among people with treatment-resistant schizophrenia: data from a systematic review and meta-analysis. Can J Psychiatry.

[4] Paul SM, Yohn SE, Popiolek M, Miller AC, Felder CC (2022). Muscarinic acetylcholine receptor agonists as novel treatments for schizophrenia. Am J Psychiatry.

[5] Marchese G, Casu G, Casti P, Spada GP, Pani L (2009). Evaluation of amphetamine-induced hyperlocomotion and catalepsy following long-acting risperidone administration in rats. Eur J Pharmacol.

[6] Brannan SK, Sawchak S, Miller AC, Lieberman JA, Paul SM, Breier A (2021). Muscarinic cholinergic receptor agonist and peripheral antagonist for schizophrenia. N Engl J Med.

[7] Mirza NR, Peters D, Sparks RG (2003). Xanomeline and the antipsychotic potential of muscarinic receptor subtype selective agonists. CNS Drug Rev.

[8] Davie BJ, Christopoulos A, Scammells PJ (2013). Development of M1 mAChR allosteric and bitopic ligands: prospective therapeutics for the treatment of cognitive deficits. ACS Chem Neurosci.

[9] (2026). Food and Drug Administration. FDA approves drug with new mechanism of action for treatment of schizophrenia. https://www.fda.gov/news-events/press-announcements/fda-approves-drug-new-mechanism-action-treatment-schizophrenia.

[10] Scarr E, Gibbons AS, Neo J, Udawela M, Dean B (2013). Cholinergic connectivity: it's implications for psychiatric disorders. Front Cell Neurosci.

[11] Lieberman JA, Javitch JA, Moore H (2008). Cholinergic agonists as novel treatments for schizophrenia: the promise of rational drug development for psychiatry. Am J Psychiatry.

[12] Yohn SE, Weiden PJ, Felder CC, Stahl SM (2022). Muscarinic acetylcholine receptors for psychotic disorders: bench-side to clinic. Trends Pharmacol Sci.

[13] Dean B, Scarr E (2020). Muscarinic M1 and M4 receptors: hypothesis driven drug development for schizophrenia. Psychiatry Res.

[14] Kring AM, Elis O (2013). Emotion deficits in people with schizophrenia. Annu Rev Clin Psychol.

[15] White FJ, Wang RY (1984). A10 dopamine neurons: role of autoreceptors in determining firing rate and sensitivity to dopamine agonists. Life Sci.

[16] Hasan AH, Abid MA (2024). Cobenfy (xanomeline-trospium chloride): a new frontier in schizophrenia management. Cureus.

[17] Jauhar S, Veronese M, Nour MM (2019). The effects of antipsychotic treatment on presynaptic dopamine synthesis capacity in first-episode psychosis: a positron emission tomography study. Biol Psychiatry.

[18] Kaul I, Sawchak S, Claxton A (2024). Efficacy of xanomeline and trospium chloride in schizophrenia: pooled results from three 5-week, randomized, double-blind, placebo-controlled, EMERGENT trials. Schizophrenia (Heidelb).

[19] Hussain JM, Arif W, Humayun S, Shoaib N, Khan SM (2025). Xanomeline‐trospium chloride (Cobenfy): a new era in managing schizophrenia—comparative effectiveness and economic challenges. Med Avd.

[20] (2026). Food and Drug Administration. FDA strengthens warning that untreated constipation caused by schizophrenia medicine clozapine (Clozaril) can lead to serious bowel problems. https://www.fda.gov/drugs/drug-safety-and-availability/fda-strengthens-warning-untreated-constipation-caused-schizophrenia-medicine-clozapine-clozaril-can.

